# Are Kenyans Likely to Use COVID-19 Self-Testing Kits? Results From a Cross-Sectional Survey

**DOI:** 10.3389/ijph.2022.1604918

**Published:** 2022-08-26

**Authors:** Griffins Manguro, Sonjelle Shilton, Sharon Omenda, Patrica Owira, Deepshikha Batheja, Abhik Banerji, Sophie Vusha Chabeda, Marleen Temmerman, Walter Jako, Joseph Ndungu, Stanley Luchters, Elena Ivanova Reipold, Guillermo Z. Martínez-Pérez

**Affiliations:** ^1^ International Centre for Reproductive Health Kenya, Mombasa, Kenya; ^2^ Foundation for Innovative New Diagnostics, Geneva, Switzerland; ^3^ Center for Disease Dynamics, Economics & Policy (CDDEP), New Delhi, India; ^4^ Department of Population Health, Aga Khan University (Kenya), Nairobi, Kenya

**Keywords:** COVID-19, survey, home diagnostics, Kenya, SARS-CoV-2 testing, self-testing

## Abstract

**Objectives:** To understand the public’s perceptions around rapid SARS-CoV-2 antigen self-testing in Kenya, including the drivers of acceptability, willingness to pay, and adherence to hygiene and prevention recommendations following a positive self-test.

**Methods:** A household-based, cross-sectional survey, using a 35-item questionnaire, was conducted in Mombasa and Taita–Taveta counties, Kenya, during August 2021. Individuals aged ≥18 years were enrolled using a stratified sampling approach.

**Results:** There were 419 participants (mean age 35.7 years). A minority (10.5%) had ever tested for SARS-CoV-2. If SARS-CoV-2 self-testing were available, 39.9% and 41.5% would be likely and very likely, respectively, to use it. If unavailable free-of-charge, 63.01% would pay for it. Multivariate analyses suggested that people in rural areas (Coefficient 0.30, 95%CI: 0.11–0.48, *p* = 0.002), aged 36–55 (Coefficient 0.21, 95%CI: 0.03–0.40, *p* = 0.023), and employed full time (Coefficient 0.32, 95%CI: 0.06–0.58, *p* = 0.016) would have more odds to adhere to recommended hygiene and prevention actions.

**Conclusion:** SARS-CoV-2 self-testing was considered acceptable. Availability of self-testing could expand access to COVID-19 testing in Kenya, particularly among rural communities who have limited access to testing, and among mildly symptomatic individuals.

## Introduction

The first case of coronavirus disease 2019 (COVID-19), which is caused by the novel strain of severe acute respiratory syndrome coronavirus 2 (SARS-CoV-2), was reported in Wuhan, China, in December 2019 [1]. Eastern Africa is among the regions where strict measures to combat the COVID-19 pandemic (i.e., quarantine, lockdowns, and travel bans) have been systematically applied, such as in Kenya, Uganda, and Rwanda, as part of these countries’ responses to curb the incidence of new COVID-19 infections [2–5].

As of February 2022, Kenya had reported 322,151 cases of COVID-19 and 5,621 deaths [6]. Vaccination against SARS-CoV-2 and ongoing testing for symptomatic cases and their close contacts are among the strategies in place in Kenya to tackle COVID-19. Due to limited laboratory capacity in Kenya, however, the number of cases might have been under-reported. Decentralized screening for diseases at community-level is an approach that has worked well with diseases such as HIV, malaria, and tuberculosis, and which could work well with COVID-19 [7–9]. Nevertheless, Kenya is a country with substantial socio-economic and geographical disparities. Populations in Kenya, and especially in the north and in most rural areas, have different opportunities to access healthcare, irrespective of whether their health needs are in relation to COVID-19 or to other conditions.

Self-sampling and self-testing for SARS-CoV-2 are innovations that have been proposed by the African Union and the European Union to increase opportunities for COVID-19 case detection [10, 11]. Self-sampling usually requires individuals to self-collect an upper respiratory tract specimen and transport it to a reference laboratory [12, 13]. This requires a good specimen transport and results communication system and hence might not be an optimal approach for the remotest areas of Kenya. On the other hand, self-testing, which does not require individuals to send their specimen to a laboratory [10, 14, 15], might be a more feasible approach, especially in a country like Kenya that already has experience with the delivery of HIV self-testing devices [16, 17].

In settings where health authorities struggle to cater for symptomatic patients’ needs, diagnostic resources must be used to ensure confirmation of positive cases and to trace-and-test the contacts of these cases. The use of rapid SARS-CoV-2 antigen-detection self-tests (hereafter, self-tests) has the potential to help asymptomatic people know their infection status when they come into contact with somebody who tests positive, when they need to travel, or where there is a need to undergo testing as required by work or school. In addition, self-testing may be useful for symptomatic cases to confirm recovery and end isolation. In alignment with the African Union recommendations on self-testing, regulatory authorities in other highly-populated resource-constrained countries such as India [18] or Brazil [19] have also approved the use of self-testing by the general public.

Self-testing could potentially relieve the pressure on the already overwhelmed Kenyan health systems and help reactivate local economies. However, despite the optimism around self-testing, a number of challenges remain, which may reduce its potential impact on case detection in Kenya. Self-tests may require that their end-users understand written instructions on their use, how to interpret results, and how to report a positive result, which could be challenging in a country where, to the best of our knowledge, self-tests developers and manufacturers have not yet explored what the public’s values and preferences of self-testing are.

There is a dearth of data from eastern Africa relating to the potential for self-testing. To guide policy and practice decisions in Kenya to enable the home-, school-, or work-based self-detection of SARS-CoV-2 infection, a thorough understanding of the Kenyan public’s values around self-testing is needed. To address this knowledge gap, a household-based survey was conducted to understand the public’s values and attitudes toward self-testing. Other specific objectives were to understand the predictors of likelihood to use self-testing; willingness to pay for self-testing; and of potential uptake of health authorities’ recommended actions that an individual should undertake following a positive self-test result.

## Methods

### Study Design, Population, and Sites

This household-based survey was conducted during August 2021 [20]. The general populations of two study sites in Kenya were approached as respondents: the coastal county of Mombasa, representing an urban area, and the county of Taita–Taveta, representing a rural area. Any individual aged 18 years or older, who was willing to provide informed consent and had no COVID-19-attributable symptoms, was eligible for participation.

Mombasa, located on the coast of Kenya, has a population of more than one million and is where the implementing organization (i.e., International Centre for Reproductive Health Kenya) is based. The rural county of Taita–Taveta, in south-eastern Kenya, was chosen as a second survey site, with the aim of accessing survey respondents whose social, economic, and cultural characteristics differ from those in urban environments such as Mombasa.

To understand whether differences based on geography and access to health resources influence willingness to use self-testing, separate population size calculations were performed for Mombasa and Taita–Taveta. It was calculated that it would be necessary to include at least 196 respondents per site to have a 95% confidence level that the real value (likelihood to use self-testing if available) is within ±7% of the measured value.

### Sampling and Enrollment of Survey Respondents

A five-pronged sampling process was used. First, the boundaries of Mombasa and Taita–Taveta were defined using Google MyMaps®. The two resulting maps were divided into 40 numbered areas. Second, using RANDOM.Org®, the two lists of numbered areas were randomly re-arranged. The first 14 areas in each list were selected as survey areas. Third, the two lists of 14 selected areas were randomly re-arranged again. Then, starting with the first area in the list, each area was assigned to a survey shift in a 1-week schedule that comprised seven morning and seven afternoon shifts. Fourth, in each area, a series of 15 households were randomly selected, and numbered, in MyMaps®. Fifth, during each shift, the surveyors would arrive in the area assigned in their schedules and, using ViewRanger®, they would walk from one selected household to the next. As a final sampling step, an individual in each visited home was randomly selected from all individuals who met the eligibility criteria for participation. The selected person was invited to give informed consent.

In cases where nobody was present in the household indicated by ViewRanger®, or if all individuals present in the home refused to participate, the surveyors would attempt to recruit a respondent in the next nearest household.

### Data Collection, Processing, and Analysis

To minimize the risk of SARS-CoV-2 transmission, data collection was performed, where possible, in the front or back yard of a household. If this was not possible, data was collected in a room of the respondent’s choice. Surveyors and respondents wore face masks and maintained physical distance.

A 35-item questionnaire guided the data collection. The instrument had four main sections: socio-demographics (e.g., nationality, age, education, gender, and ethnic identity); perception of risk of COVID-19 and previous experiences with COVID-19 testing; acceptability of and willingness to use and pay for a self-test; and hygiene and prevention actions following a self-test result.

The questionnaire was developed based on previous FIND-supported inquiries into communities’ values for self-testing for hepatitis C virus [21]. It was written in English and translated into Swahili. Both versions were pre-piloted in Mombasa. When the final English and Swahili versions were considered acceptable, these were developed in KoBoToolbox®, re-tested during the training of surveyors in the study sites, and made accessible via the KoBoCollect® app.

The surveyors collected data using the tablet-enabled KoBoCollect® app, in the respondent’s language of choice, and submitted the responses immediately to KoBoToolbox®. Data collected from the respondents did not include personal identifiers.

Data collection in Mombasa and Taita–Taveta was conducted concurrently. Once the survey activities were complete in both sites, the English and Swahili datasets were exported into MS-Excel®, merged, and cleaned for analysis. Bivariate and multivariate descriptive and inferential analyses were conducted using STATA v.14® (StataCorp LLC.).

The main primary outcomes for analysis were: likelihood to use a self-test if available in Kenya; willingness to pay for a self-test device; and potential compliance with recommended actions following a positive self-test result (i.e., wear face mask, warn close contacts, report the result, and self-isolate). Univariate, bivariate and multivariate regression analyses were performed to explore significant associations between the primary outcomes and respondents’ socio-demographic characteristics, risk perceptions, and experiences with conventional COVID-19 testing.

Those variables that were found by the bivariate analyses as significantly associated with the primary outcomes, i.e., at a *p*-value < 0.05, were entered into the multivariate regression model. A logistic regression model was used to identify associations between likelihood to use and willingness to pay for a self-test, and their potential predictors. Ordinary least squares (OLS) regression was used to identify predictors of compliance with recommended actions following a positive self-test result through the construction of an index to identify predictors for appropriate actions taken after testing positive.

### Ethics Considerations

All respondents signed two copies of the consent documents and kept one signed copy. Each respondent received a small bag containing face masks and hand sanitizers as a token of appreciation for their participation. This research received ethical clearance from the Amref Health Africa Ethics and Scientific Review Committee (Ref.: P1011/2011) and the Kenya National Commission for Science, Technology and Innovation (License: NACOSTI/P/21/12458).

## Results

### Participants’ Characteristics

A total of 419 people (266 women, 151 men, and 2 non-binary) participated (Table 1). There were 209 respondents from Mombasa and 210 from Taita–Taveta. The mean respondents’ age in Taita–Taveta was 39.3 years (SD 13.75) for women and 39.6 years (SD 14.4) for men, compared with 31.2 years (SD 9.48) for women and 33.5 years (SD 10.8) for men in Mombasa. Mombasa had a higher combined proportion of men and women who had completed secondary school than Taita–Taveta; 38.6% of women and 29.4% of men in Taita–Taveta, compared with 32.5% of women and 47.0% of men in Mombasa. Taita–Taveta had a higher unemployment rate than Mombasa. The ethnicities most represented in the sample were Luo (6.2%), Taita (43.91%), and Kamba (7.4%).

**TABLE 1:** Participants’ sociodemographic characteristics (Mombasa and Taita-Taveta, Kenya. 2021).

	Rural (Taita-Taveta)			Urban (Mombasa)		Total (Rural and Urban)			
	Female (*n* = 140)	Male (*n* = 68)	Non- binary (*n* = 2)	Female (*n* = 126)	Male (*n* = 83)	Female (*n* = 266)	Male (*n* = 151)	Non- binary (*n* = 2)	Total (*n* = 419)
Mean Age (Std. Deviation) in Years	39.252 (13.756)	39.618 (14.354)	29 (4.24)	31.238 (9.478)	33.506 (10.836)	35.442 (12.551)	36.258 (12.866)	29 (4.24)	35.705 (12.63)
Age groups									
18–25	27 (19.29%)	10 (14.71%)	0 (0.00%)	45 (35.71%)	24 (28.92%)	72 (27.07%)	34 (22.52%)	0 (0.00%)	106 (25.30%)
26–35	39 (27.86%)	23 (33.82%)	2 (100.0%)	49 (38.89%)	32 (38.55%)	88 (33.08%)	55 (36.42%)	2 (100.0%)	145 (34.61%)
36–45	29 (20.71%)	12 (17.65%)	0 (0.00%)	23 (18.25%)	15 (18.07%)	52 (19.55%)	27 (17.88%)	0 (0.00%)	79 (18.85%)
46–55	19 (13.57%)	12 (17.65%)	0 (0.00%)	4 (3.17%)	6 (7.23%)	23 (8.65%)	18 (11.92%)	0 (0.00%)	41 (9.79%)
56–65	21 (15.00%)	8 (11.76%)	0 (0.00%)	5 (3.97%)	6 (7.23%)	26 (9.77%)	14 (9.27%)	0 (0.00%)	40 (9.55%)
65 and above	5 (3.57%)	3 (4.41%)	0 (0.00%)	0 (0.00%)	0 (0.00%)	5 (1.88%)	3 (1.99%)	0 (0.00%)	8 (1.91%)
Level of education									
None	2 (1.43%)	0 (0.00%)	0 (0.00%)	3 (2.38%)	2 (2.41%)	5 (1.88%)	2 (1.32%)	0 (0.00%)	7 (1.67%)
Primary	51 (36.43%)	23 (33.82%)	2 (100.0%)	46 (36.51%)	26 (31.33%)	97 (36.47%)	49 (32.45%)	2 (100.0%)	148 (35.32%)
Secondary	54 (38.57%)	20 (29.41%)	0 (0.00%)	41 (32.54%)	39 (46.99%)	95 (35.71%)	59 (39.07%)	0 (0.00%)	154 (36.75%)
College/Vocational Training	26 (18.57%)	19 (27.94%)	0 (0.00%)	27 (21.43%)	7 (8.43%)	53 (19.92%)	26 (17.22%)	0 (0.00%)	79 (18.85%)
University Degree	7 (5.00%)	6 (8.82%)	0 (0.00%)	8 (6.35%)	9 (10.84%)	15 (5.64%)	15 (9.93%)	0 (0.00%)	30 (7.16%)
Most represented ethnic identities (out of 44 self-reported ethnicities)									
Kamba	10 (7.14%)	4 (5.88%)	0 (0.00%)	12 (9.52%)	5 (6.02%)	22 (8.27%)	9 (5.96%)	0 (0.00%)	31 (7.40%)
Luo	3 (2.14%)	1 (1.47%)	0 (0.00%)	15 (11.90%)	7 (8.43%)	18 (6.76%)	8 (4.64%)	0 (0.00%)	26 (6.20%)
Taita	105 (75.00%)	55 (80.88%)	2 (100.00%)	13 (10.32%)	10 (12.05%)	118 (33.36%)	65 (43.05%)	2 (100.00%)	185 (44.15%)
Employment Status									
Unemployed	51 (36.43%)	15 (22.06%)	0 (0.00%)	43 (34.13%)	17 (20.48%)	94 (35.34%)	32 (21.19%)	0 (0.00%)	126 (30.07%)
Student	5 (3.57%)	0 (0.00%)	0 (0.00%)	3 (2.38%)	5 (6.02%)	8 (3.01%)	5 (3.31%)	0 (0.00%)	13 (3.10%)
Employed, Part-time	12 (8.57%)	11 (16.18%)	1 (50.00%)	7 (5.56%)	16 (19.28%)	19 (7.14%)	27 (17.88%)	1 (50.00%)	47 (11.22%)
Employed, Full-time	15 (10.71%)	7 (10.29%)	0 (0.00%)	17 (13.49%)	15 (18.07%)	32 (12.03%)	22 (14.57%)	0 (0.00%)	54 (12.89%)
Self-employed, part time	13 (9.29%)	4 (5.88%)	0 (0.00%)	16 (12.70%)	8 (9.64%)	29 (10.90%)	12 (7.95%)	0 (0.00%)	41 (9.79%)
Self-employed, full time	43 (30.71%)	30 (44.12%)	1 (50.00%)	40 (31.75%)	21 (25.30%)	83 (31.20%)	51 (33.77%)	1 (50.00%)	135 (32.22%)
Retired on a pension	1 (0.71%)	1 (1.47%)	0 (0.00%)	0 (0.00%)	1 (1.20%)	1 (0.38%)	2 (1.32%)	0 (0.00%)	3 (0.72%)

### Experience With Provider-Initiated COVID-19 Testing

Only 30 (7.2%) respondents believed they were not at risk of severe COVID-19 disease; 117 (28%) believed they were at high risk of severe disease (Table 2). Overall, 368 (87%) respondents had never tested for COVID-19. Just 44 (10.5%) respondents had tested at least once. Among these, most (*n* = 26) had tested only once. Women and men in Mombasa were more likely to have tested compared with their counterparts in Taita-Taveta; 8.8% of women and 19.3% of men in Mombasa had been tested, compared with 9.3% of women and 5.9% of men in Taita–Taveta. Those who had previously tested at least once had received a test an average of 4.5 (SD 4.2) months before. Half of those who had ever received a COVID-19 test rated their experience as inconvenient (27.3%) or very inconvenient (22.7%). Half of them (*n* = 22 out of 44) paid an average cost of 6.9 USD (SD 9.5) for their most recent test.

**TABLE 2:** Participants’ previous experience with COVID-19 testing (Mombasa and Taita-Taveta, Kenya. 2021).

	Rural (Taita-Taveta)			Urban (Mombasa)		Total (Rural and Urban)			
	Female (*n* = 140)	Male (*n* = 68)	Non- binary (*n* = 2)	Female (*n* = 126)	Male (*n* = 83)	Female (*n* = 266)	Male (*n* = 151)	Non- binary (*n* = 2)	Total (*n* = 419)
Perception of risk of COVID-19									
No risk	7 (5.00%)	6 (8.82%)	0 (0.00%)	9 (7.14%)	8 (9.64%)	16 (6.02%)	14 (9.27%)	0 (0.00%)	30 (7.16%)
Low risk	33 (23.57%)	15 (22.06%)	0 (0.00%)	34 (26.98%)	29 (34.94%)	67 (25.19%)	44 (29.14%)	0 (0.00%)	111 (26.49%)
Mild risk	36 (25.71%)	12 (17.65%)	2 (100.00%)	27 (21.43%)	10 (12.05%)	63 (23.68%)	22 (14.57%)	2 (100.00%)	87 (20.76%)
Moderate risk	27 (19.29%)	18 (26.47%)	0 (0.00%)	16 (12.70%)	13 (15.66%)	43 (16.17%)	31 (20.53%)	0 (0.00%)	74 (17.66%)
High risk	37 (26.43%)	17 (25.00%)	0 (0.00%)	40 (31.75%)	23 (27.71%)	77 (28.95%)	40 (26.49%)	0 (0.00%)	117 (27.92%)
Number of times have you felt that you needed testing for COVID-19 but you could NOT access testing									
Never	79 (56.43%)	35 (51.47%)	1 (50.00%)	65 (51.59%)	41 (49.40%)	144 (54.14%)	76 (50.33%)	1 (50.00%)	221 (52.74%)
Once	19 (13.57%)	9 (13.24%)	1 (50.00%)	19 (15.08%)	9 (10.84%)	38 (14.29%)	18 (11.92%)	1 (50.00%)	57 (13.60%)
Twice	17 (12.14%)	12 (17.65%)	0 (0.00%)	5 (3.97%)	6 (7.23%)	22 (8.27%)	18 (11.92%)	0 (0.00%)	40 (9.55%)
Three times	2 (1.43%)	1 (1.47%)	0 (0.00%)	7 (5.56%)	5 (6.02%)	9 (3.38%)	6 (3.97%)	0 (0.00%)	15 (3.58%)
More than three times	17 (12.14%)	11 (16.18%)	0 (0.00%)	27 (21.43%)	20 (24.10%)	44 (16.54%)	31 (20.53%)	0 (0.00%)	75 (17.90%)
Not sure/cannot remember	6 (4.29%)	0 (0.00%)	0 (0.00%)	3 (2.38%)	2 (2.41%)	9 (3.38%)	2 (1.32%)	0 (0.00%)	11 (2.63%)
At least once	55 (39.29%)	33 (48.53%)	1 (50.00%)	58 (46.03%)	40 (48.19%)	113 (42.48%)	73 (48.34%)	1 (50.00%)	187 (44.63%)
Number of times you tested for COVID-19									
Never	123 (87.86%)	62 (91.18%)	2 (100.0%)	114 (90.48%)	67 (80.72%)	237 (89.10%)	129 (85.43%)	2 (100.0%)	368 (87.83%)
Once	10 (7.14%)	1 (1.47%)	0 (0.00%)	6 (4.76%)	9 (10.84%)	16 (6.02%)	10 (6.62%)	0 (0.00%)	26 (6.21%)
Twice	2 (1.43%)	3 (4.41%)	0 (0.00%)	3 (2.38%)	3 (3.61%)	5 (1.88%)	6 (3.97%)	0 (0.00%)	11 (2.63%)
Three times	0 (0.00%)	0 (0.00%)	0 (0.00%)	0 (0.00%)	1 (1.20%)	0 (0.00%)	1 (0.66%)	0 (0.00%)	1 (0.24%)
More than three times	1 (0.71%)	0 (0.00%)	0 (0.00%)	2 (1.59%)	3 (3.61%)	3 (1.13%)	3 (1.99%)	0 (0.00%)	6 (1.43%)
Not sure/cannot remember	4 (2.86%)	2 (2.94%)	0 (0.00%)	1 (0.79%)	0 (0.00%)	5 (1.88%)	2 (1.32%)	0 (0.00%)	7 (1.67%)
At least once ^a^	13 (9.29%)	4 (5.88%)	0 (0.00%)	11 (8.73%)	16 (19.28%)	24 (9.02%)	20 (13.25%)	0 (0.00%)	44 (10.50%)
Time last test was received (in months) ^a^									
Mean	6.67	4	0	4.3	3.38	5.42	3.47	0	4.45
Standard Deviation	6.5	2	0	2.56	3	5.15	2.83	0	4.22
Perception of convenience of last test ^a^									
Very convenient	2 (15.38%)	0 (0.00%)	0 (0.00%)	0 (0.00%)	3 (18.75%)	2 (8.33%)	3 (15.00%)	0 (0.00%)	5 (11.36%)
Convenient	2 (15.38%)	2 (50.00%)	0 (0.00%)	3 (27.27%)	3 (18.75%)	5 (20.83%)	5 (25.00%)	0 (0.00%)	10 (22.73%)
Neutral	2 (15.38%)	0 (0.00%)	0 (0.00%)	3 (27.27%)	2 (12.50%)	5 (20.83%)	2 (10.00%)	0 (0.00%)	7 (15.91%)
Inconvenient	5 (38.46%)	1 (25.00%)	0 (0.00%)	3 (27.27%)	3 (18.75%)	8 (33.33%)	4 (20.00%)	0 (0.00%)	12 (27.27%)
Very inconvenient	2 (15.38%)	1 (25.00%)	0 (0.00%)	2 (18.18%)	5 (31.25%)	4 (16.67%)	6 (30.00%)	0 (0.00%)	10 (22.73%)
Payment made for last COVID-19 test (in USD) ^a^									
Paid for the test	4 (30.77%)	3 (75.00%)	0 (0.00%)	6 (54.55%)	9 (56.25%)	10 (41.67%)	12 (60.00%)	0 (0.00%)	22 (50.00%)
Mean	6	5.76	0	5.56	6.61	5.74	6.399	0	6.9
Std. Deviation	7.26	9.56	0	7.08	12.05	6.75	11.06	0	9.15

a Denominators of variables “Time last test was received,” “Perception of convenience of last test,” and “Payment for last COVID-19 test (in USD)” are those who answered having tested for COVID-19 “At least once”.

In total, 187 (44.6%) respondents said they had wanted a COVID-19 test at least once but were unable to because they could not get access to testing (Table 2). The proportion of people feeling they could not access testing when needed was higher among females in Mombasa (46%) than among females in Taita-Taveta (39%). There was, however, slight difference in responses among male respondents from the two study sites.

### Knowledge and Acceptability of COVID-19 Self-Testing

We asked respondents if they were aware of the availability of self-testing for a variety of conditions. HIV, malaria, and pregnancy self-testing were the most mentioned self-testing devices (Table 3). When asked specifically about SARS-CoV-2 self-testing, only 9.2% of the overall population surveyed knew about it. This proportion was much higher in Mombasa (17.9% and 17.1% for females and males, respectively) than in Taita-Taveta (0.7% and 1.5%, respectively).

**TABLE 3:** Acceptability of self-testing (Mombasa and Taita-Taveta, Kenya. 2021).

	Rural (Taita-Taveta)			Urban (Mombasa)		Total (Rural and Urban)			
	Female (*n* = 140)	Male (*n* = 68)	Non- binary (*n* = 2)	Female (*n* = 126)	Male (*n* = 83)	Female (*n* = 266)	Male (*n* = 151)	Non- binary (*n* = 2)	Total (*n* = 419)
Awareness of other self-testing devices									
Infectious diseases									
HIV	102 (72.86%)	56 (82.35%)	1 (50.00%)	97 (78.86%)	69 (84.15%)	199 (75.67%)	125 (83.33%)	1 (50.00%)	325 (78.31%)
Malaria	58 (41.43%)	27 (39.71%)	1 (50.00%)	68 (55.28%)	45 (54.88%)	126 (47.91%)	72 (48.00%)	1 (50.00%)	199 (47.95%)
Syphilis	1 (0.71%)	1 (1.47%)	0 (0.00%)	2 (1.63%)	0 (0.00%)	3 (1.14%)	1 (0.67%)	0 (0.00%)	4 (0.96%)
Ulcer (Helicobacter Pylori)	0 (0.00%)	1 (1.47%)	0 (0.00%)	2 (1.63%)	0 (0.00%)	2 (0.76%)	1 (0.67%)	0 (0.00%)	3 (0.72%)
Human Papillomavirus	0 (0.00%)	0 (0.00%)	0 (0.00%)	0 (0.00%)	0 (0.00%)	0 (0.00%)	0 (0.00%)	0 (0.00%)	0 (0.00%)
SARS-CoV-2	1 (0.71%)	1 (1.47%)	0 (0.00%)	22 (17.89%)	14 (17.07%)	23 (8.75%)	15 (10.00%)	0 (0.00%)	38 (9.16%)
Hepatitis C virus	1 (0.71%)	0 (0.00%)	0 (0.00%)	1 (0.81%)	0 (0.00%)	2 (0.76%)	0 (0.00%)	0 (0.00%)	2 (0.48%)
Non-infectious conditions									
Hypertension	34 (24.29%)	13 (19.12%)	0 (0.00%)	14 (11.38%)	13 (15.85%)	48 (18.25%)	26 (17.33%)	0 (0.00%)	74 (17.83%)
Diabetes/Glycaemia	39 (27.86%)	25 (36.76%)	0 (0.00%)	31 (25.20%)	25 (30.49%)	70 (26.62%)	50 (33.33%)	0 (0.00%)	120 (28.92%)
Pregnancy	119 (85.00%)	45 (66.18%)	2 (100.0%)	111 (90.24%)	50 (60.98%)	230 (87.45%)	95 (63.33%)	2 (100.0%)	327 (78.80%)
Substances (alcohol, cocaine, marijuana etc.)	11 (7.86%)	3 (4.41%)	0 (0.00%)	7 (5.69%)	2 (2.44%)	18 (6.84%)	5 (3.33%)	0 (0.00%)	23 (5.54%)
Agreement with the concept of home COVID-19 self-testing									
Yes	128 (91.43%)	63 (92.65%)	1 (50%)	115 (91.27%)	73 (87.95%)	243 (91.35%)	136 (90.07%)	1 (50.00%)	380 (90.69%)
No	9 (6.43%)	3 (4.41%)	0 (0.00%)	6 (4.76%)	9 (10.84%)	15 (5.64%)	12 (7.95%)	0 (0.00%)	27 (6.44%)
Not sure/cannot say	3 (2.14%)	2 (2.94%)	1 (50.00%)	5 (3.97%)	1 (1.20%)	8 (3.01%)	3 (1.99%)	1 (50.00%)	12 (2.86%)
Willingness to pay for a self-testing device if needed									
Reported willingness to pay for a COVID-19 self-test	77 (55.00%)	39 (57.35%)	1 (50.00%)	89 (70.63%)	58 (69.88%)	166 (62.41%)	97 (64.24%)	1 (50.00%)	264 (63.01%)
Mean amount (in USD) participants willing to pay for a COVID-19 self-test	0.669	0.6	1.2	0.59	0.67	0.62	0.65	1.2	0.63
Standard deviation	1.14	0.59	0	1.15	0.96	1.14	0.83	0	1.03
Likelihood of using COVID-19 self-testing if needed, if available in Kenya									
Very unlikely	2 (1.43%)	3 (4.41%)	0 (0.00%)	5 (3.97%)	4 (4.82%)	7 (2.63%)	7 (4.64%)	0 (0.00%)	14 (3.34%)
Unlikely	8 (5.71%)	2 (2.94%)	0 (0.00%)	1 (0.79%)	1 (1.20%)	9 (3.38%)	3 (1.99%)	0 (0.00%)	12 (2.86%)
Neutral	24 (17.14%)	14 (20.59%)	1 (50.00%)	10 (7.94%)	3 (3.61%)	34 (12.78%)	17 (11.26%)	1 (50.00%)	52 (12.41%)
Likely	63 (45.00%)	25 (36.76%)	1 (50.00%)	44 (34.92%)	34 (40.96%)	107 (40.23%)	59 (39.07%)	1 (50.00%)	167 (39.86%)
Very likely	43 (30.71%)	24 (35.29%)	0 (0.00%)	66 (52.38%)	41 (49.40%)	109 (40.98%)	65 (43.05%)	0 (0.00%)	174 (41.53%)
Mean (SD)	3.9 (0.91)	3.95 (1.04)	3.5 (0.7)	4.3 (0.95)	4.2 (0.96)	4.13 (0.94)	4.13 (1.01)	3.5 (0.7)	4.1 (0.96)

The majority of respondents (90.69%) agreed with the concept or idea of people being able to self-test at home for COVID-19 (Table 3). There was slight difference in the proportion that agreed with the concept of self-testing between women and men, and between those who lived in urban versus rural areas. As per the univariate analyses, agreement with the concept of self-testing was significantly associated with being from an urban area (*p* < 0.001), feeling at no risk (*p* < 0.001) or at mild risk (*p* = 0.024) of COVID-19 disease, and not having had access to COVID-19 testing when needed (*p* = 0.028).

If self-testing were available in Kenya, 167 (39.9%) of all respondents said they would be likely to use self-testing, and 174 (41.5%) would be very likely to use self-testing (Table 3). The proportion of female and male respondents who reported being likely or very likely to use SARS-CoV-2 self-testing if they needed it was higher in Mombasa than in Taita-Taveta. In Mombasa, 110 (87.3%) female and 75 (90.36%) male respondents would use self-testing, compared with 106 (75.71%) females and 49 (72.05%) males in Taita–Taveta.

**FIGURE 1:**
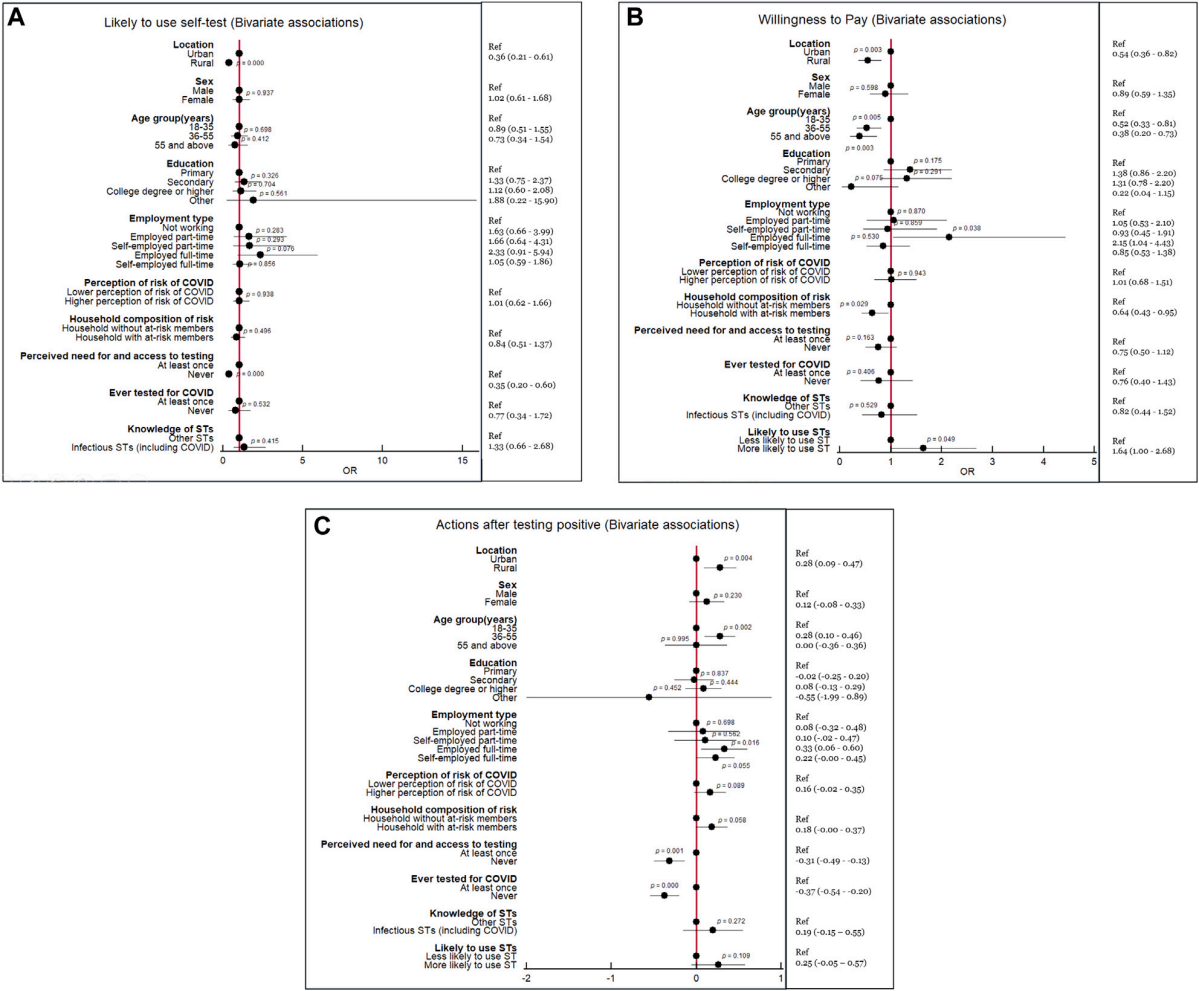
Significant associations detected in bivariate analyses of primary outcomes (*P* values in the forest plot, Odds ratio and 95% Confidence Intervals in right column) (Mombasa and Taita-Taveta, Kenya. 2021).

Univariate inferential analyses showed that being very likely or likely to use self-testing was significantly associated with being from an urban (*p* < 0.001) or rural area (*p* < 0.001) and with being retired and on a pension (*p* = 0.034). Being very unlikely or unlikely to use a self-test was associated with feeling at mild risk of COVID-19 (*p* < 0.01) and being satisfied with the most recent testing taken at a facility (*p* = 0.045). The bivariate analysis showed that people who felt the need to test but were unable to access conventional testing were less likely to use self-testing (Odds ratio (OR): 0.35, 95% Confidence Interval (CI): 0.20–0.60, *p* < 0.001), and that people from rural areas were less likely than people from urban areas to use self-testing (OR = 0.36, 95%CI: 0.21–0.61, *p* < 0.001) (Figure 1A).

### Willingness to Pay

If self-testing were available in Kenya, 264 (63.0%) of all respondents would be willing to pay for it if they needed it and it were not provided free-of-charge by the health system (Table 3). The proportion of female and male respondents who stated they would be willing to pay was higher in Mombasa (70.6% females, 69.88% males) than in Taita-Taveta (55.0% females, 57.4% males).

**FIGURE 2:**
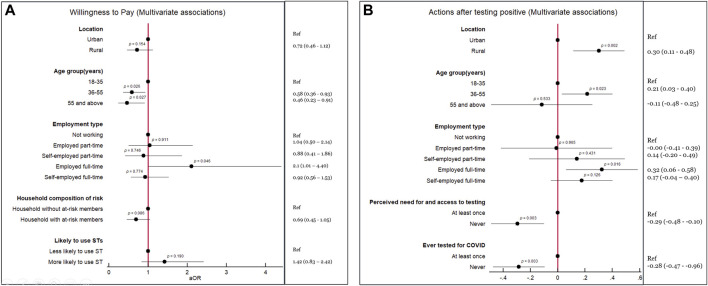
Significant associations detected in multivariate analyses of primary outcomes (*P* values in the forest plot, Odds ratio and/or Coefficients and 95% Confidence Intervals in right column) (Mombasa and Taita-Taveta, Kenya. 2021).

As per univariate analyses, willingness to pay for a self-testing device was significantly associated with being from rural (*p* = 0.003) and urban (*p* = 0.003) areas; being in age groups 18–35 (*p* < 0.001), 36–55 (*p* = 0.004), and >55 years (*p* = 0.004); and with being in full-time employment (*p* = 0.04) (Figure 1B). However, the multivariate regression model only confirmed that respondents in the age grouping 36–35 (OR 0.58, 95% CI: 0.336–0.93, *p* = 0.026) and those older than 55 years old (OR 0.46, 95% CI 0.23–0.91, *p* = 0.027) would be less likely to be willing to pay for a self-test (Figure 2A).

### Actions Upon Self-Testing for COVID-19

Respondents in Mombasa expressed that they would be more likely to report a positive self-test result to a health facility (86.40% females, 82.93% males), whereas those in Taita-Taveta were more likely to report to a community health volunteer or a village health worker (73.57% females, 70.59% males) (Table 4).

**TABLE 4:** Steps in the event that a respondent self-tests positive (Mombasa and Taita-Taveta, Kenya. 2021).

	Rural (Taita-Taveta)			Urban (Mombasa)		Total (Rural and Urban)			
	Female (*n* = 140)	Male (*n* = 68)	Non- binary (*n* = 2)	Female (*n* = 126)	Male (*n* = 83)	Female (*n* = 266)	Male (*n* = 151)	Non- binary (*n* = 2)	Total (*n* = 419)
Preferred channels to report a positive COVID-19 result if participants used a COVID-19 self-test									
By going in person to my clinic/hospital (i.e., directly to a healthcare worker)	74 (52.86%)	43 (63.24%)	2 (100.0%)	108 (86.40%)	68 (82.93%)	182 (68.68%)	111 (74.00%)	2 (100.0%)	295 (70.74%)
Through community/village health workers	103 (73.57%)	48 (70.59%)	1 (50.00%)	68 (54.40%)	37 (45.12%)	171 (64.53%)	85 (56.67%)	1 (50.00%)	257 (61.63%)
Through NGO/CSO extension workers^a^	27 (19.29%)	8 (11.76%)	0 (0.00%)	18 (14.40%)	13 (15.85%)	45 (16.98%)	21 (14.00%)	0 (0.00%)	66 (15.83%)
Through phone call (e.g., hotline, toll free line, COVID line, nearest COVID-19 centre...)	68 (48.57%)	33 (48.53%)	0 (0.00%)	59 (47.20%)	40 (48.78%)	127 (47.92%)	73 (48.67%)	0 (0.00%)	200 (47.96%)
Through internet (e.g., website, phone application)	11 (7.86%)	4 (5.88%)	0 (0.00%)	6 (4.80%)	3 (3.66%)	17 (6.42%)	7 (4.67%)	0 (0.00%)	24 (5.76%)
Through a pharmacist	21 (15.00%)	5 (7.35%)	0 (0.00%)	15 (12.00%)	6 (7.32%)	36 (13.58%)	11 (7.33%)	0 (0.00%)	47 (11.27%)
Through a teacher/mentor/professor	3 (2.14%)	0 (0.00%)	0 (0.00%)	1 (0.80%)	2 (2.44%)	4 (1.51%)	2 (1.33%)	0 (0.00%)	6 (1.44%)
Through an employer/boss	0 (0.00%)	2 (2.94%)	0 (0.00%)	8 (6.40%)	7 (8.54%)	8 (3.02%)	9 (6.00%)	0 (0.00%)	17 (4.08%)
If you used a COVID-19 self-test and its result were POSITIVE, would you do the following									
Communicate/report your result to your clinic/hospital and/or to the COVID hotline	135 (96.43%)	65 (95.59%)	2 (100.00%)	116 (92.06%)	72 (86.75%)	251 (94.36%)	137 (90.73%)	2 (100.00%)	390 (93.08%)
Go in person to your clinic/hospital to get post-testing counselling from a healthcare professional	134 (95.71%)	64 (94.12%)	2 (100.00%)	115 (91.27%)	76 (91.57%)	249 (93.61%)	140 (92.72%)	2 (100.00%)	391 (93.32%)
Self-isolate	133 (95.00%)	65 (95.59%)	2 (100.00%)	120 (95.24%)	79 (95.18%)	253 (95.11%)	144 (95.36%)	2 (100.00%)	399 (95.23%)
Identify and warn/call your close contacts	136 (97.14%)	65 (95.59%)	1 (50.00%)	111 (88.80%)	69 (83.13%)	247 (93.21%)	134 (88.74%)	1 (50.00%)	382 (91.39%)
Inform your employer	61 (73.49%)	41 (78.85%)	0 (0.00%)	67 (88.16%)	51 (86.44%)	128 (80.50%)	92 (82.88%)	0 (0.00%)	220 (80.88%)

a CSO, civil society organization; NGO, non-governmental organization.

Almost all respondents (*n* = 390, 93.08%) reported that they would report a positive self-test, that they would self-isolate (*n* = 399, 95.23%), and that they would warn their close contacts (*n* = 382, 91.4%) (Table 4). Most of the respondents who were employed (*n* = 220 out of 272, 80.8%) reported that they would communicate the positive result to their employer.

As per the univariate inferential analysis, there was an association between agreeing with the concept of self-testing and stating an intention to communicate a result (*p* = 0.008) and warn contacts (*p* < 0.001). There was also an association between respondents being willing to pay for a self-test and stating that they would not discontinue wearing a face mask after receiving a negative result (in the presence of symptoms) (*p* < 0.001). There were associations between respondents expressing willingness to weekly self-test and stating that they would attend a clinic to request post-test counseling (*p* = 0.005); would warn close contacts (*p* = 0.04); and would not stop practicing social distancing (in the presence of symptoms) even if they self-tested negative (*p* = 0.024) (Figure 1C). The OLS model confirmed that people in rural areas (Coefficient 0.30, 95% CI: 0.11–0.48, *p* = 0.002), people in the age grouping 36–55 (Coefficient 0.21, 95% CI: 0.03–0.40, *p* = 0.023), and people employed full time (Coefficient 0.32, 95% CI: 0.06–0.58, *p* = 0.016) would have more odds to adhere to recommended actions following a positive self-test. As per the OLS, people who never felt they could not access testing when they needed it (Coefficient –0.29, 95% CI: –0.48 to −0.10, *p* = 0.003) and those who had never tested for COVID-19 before (Coefficient −0.23, 95% CI: –0.47 to −0.96, *p* = 0.003) would have less odds to adhere to recommended hygiene and prevention actions (Figure 2B).

## Discussion

To understand people’s values in relation to SARS-CoV-2 self-testing in a resource-limited setting such as Kenya, we surveyed 419 respondents in two geographically and socio-economically distinct counties. About half of individuals surveyed (44.73%) stated they had wanted to test for COVID-19 at least once but had been unable to access testing. Only around one tenth (9.16%) of respondents had heard of a SARS-CoV-2 self-test, but a clear majority stated they would be likely (39.86% of the sample) or very likely (41.53%) to use it if they needed and if these were available in Kenya. If there was a charge for the self-test, 63.01% of respondents would pay for it, and the average acceptable cost was US$ 0.63.

By February 2022, more than 322,151 SARS-CoV-2 infections had been reported in Kenya [6]. Most of the individuals infected in Kenya, as in many other regions of the world, lived in cities, with Nairobi and Mombasa accounting for more than half of all infections. In developing countries, higher rates of infection in urban areas are likely because of the many informal settlements in these areas, where it is easy for the virus to spread [22]. Only about one tenth (10.50%) of our survey respondents had ever tested for SARS-CoV-2, which is likely to be an indication of generally low testing rates in Kenya, compared with 34.9% of respondents who reported ever having a test in a similar survey conducted in Indonesia [23]. The most commonly used testing method in Kenya is real-time polymerase chain reaction (RT-PCR) testing of upper respiratory tract specimens. Most testing is performed at health facilities and is generally inaccessible to the majority of the public. As of September 2021, there were just 52 testing centers in the whole of Kenya [24], servicing a population of more than 53 million people [25].

In our study, about half of those who have ever been tested said it was “inconvenient” (27.27%) or “very inconvenient” (22.73%), whereas in a similar survey in Indonesia, 30% and 1.2% of respondents reported their testing was “inconvenient” or “very inconvenient,” respectively [23]. There may be a number of reasons for these responses in Kenya. One reason could be the long lines at testing centers, which are limited in number and have to cope with a considerable number of individuals needing to get tested, as well as the long turnaround times for the communication of results. Expanded availability to COVID-19 testing through self-testing could help overcome such facility-based inconveniences.

Many survey respondents had heard of HIV self-testing, but just a few (9.16%) had heard of SARS-CoV-2 self-testing. In Kenya, HIV self-testing has been provided in the public sector since 2017, and HIV self-testing kits are distributed free-of-charge in public facilities and at a reduced price in private facilities [26]. Studies have shown that HIV self-testing is quite acceptable in Kenya, and it has helped to increase testing rates and enhance the early diagnosis of HIV [16, 27, 28]. In a study involving both men and women in Kenya, virtually all respondents stated that using the HIV self-testing kits was simple, empowering, and reduced the anxiety associated with waiting for HIV tests at clinics [16]. Due to this reported public’s awareness of HIV self-testing as an acceptable and empowering technology, it is perhaps not surprising, then, that despite the fact that the majority of our survey respondents were unaware of SARS-CoV-2 self-testing, many of them indicated they would use it if it were available. The high acceptability of SARS-CoV-2 self-testing in our survey is aligned with findings of similar acceptability studies in Germany [14], Greece [15], or Indonesia [23].

An important finding of our survey was that the majority of respondents would be willing to pay for a SARS-CoV-2 self-test if it were not provided free-of-charge by health authorities. The willingness to pay was only significantly associated with respondents in the age groups 18–35 (*p* < 0.001), 36–55 (*p* = 0.004), and >55 years (*p* = 0.003); and being employed full-time (*p* = 0.04). The regression analysis only confirmed that rural area inhabitants may be less likely to pay for self-testing. These factors are similar to those factors that have been reported from other studies in the region and which influence the willingness to pay for health services in general [29–31]. The reported mean amount that respondents were willing to spend for a SARS-CoV-2 self-test was US$ 0.6, equivalent to 60 Kenyan shillings, which is half than what respondents in the Indonesian survey on SARS-CoV-2 self-testing were willing to spend (i.e., average of US$ 1.4) [23].

Another finding worth noting is that, in this study, the majority of respondents said they would report a positive result to a health facility or to community health structures and that they would take the necessary precautions to limit exposure to others, including self-isolation and wearing a face mask. This indicates that the community has an adequate predisposition to do what health authorities in other settings [11, 18, 19] recommend if someone self-tests positive for SARS-CoV-2. To guarantee that self-testing is effective in the overall approach to COVID-19, a suitable response from health authorities—including the provision of social safety nets to the most vulnerable — to a positive result is critical.

A major limitation of our study was that we asked questions about the potential value and acceptability of a putative SARS-CoV-2 self-test, instead of actually distributing a self-test kit that respondents could try. As a result, the study could be subject to social desirability bias, with respondents giving responses that they thought are most socially acceptable, but which may or may not reflect what they would do if the test were indeed available.

In conclusion, this study demonstrates that SARS-CoV-2 self-testing is acceptable among both urban and rural Kenyan populations and that it would be beneficial to expand the existing COVID-19 testing modalities. The findings of our survey, the first of its kind in the Eastern African region, have contributed to the body of evidence supporting the recent release, by the World Health Organization, of guidance for the use of SARS-CoV-2 antigen-detection rapid diagnostic tests for COVID-19 self-testing [29]. Policymakers should consider ways to make it easier for people to access self-testing, to report a positive self-test, and to link to post-self-test counseling and care. Self-testing kits should be priced within the price range of HIV or pregnancy self-test kits when marketing them, as this might increase their acceptability and use and, ultimately, help them to empower the population to become agents in COVID-19 case detection and control.
